# *In situ* X-ray diffraction investigation of electric-field-induced switching in a hybrid improper ferroelectric

**DOI:** 10.1107/S1600576721001096

**Published:** 2021-03-18

**Authors:** Gabriel Clarke, Chris Ablitt, John Daniels, Stefano Checchia, Mark S. Senn

**Affiliations:** a Department of Chemistry, University of Warwick, Coventry, United Kingdom; b School of Materials Science and Engineering, UNSW Sydney, Kensington 2052, Australia; c ESRF, The European Synchrotron, 38000, Grenoble, France

**Keywords:** hybrid improper ferroelectrics, *in situ* diffraction, Ruddlesden–Popper

## Abstract

Improper ferroelectric mechanisms are increasingly under investigation for their potential to expand the current catalogue of functional materials whilst promoting couplings between ferroelectricity and other technologically desirable properties such as ferromagnetism. This work presents the results of an *in situ* synchrotron X-ray diffraction experiment performed on samples of Ca_2.15_Sr_0.85_Ti_2_O_7_ in an effort to elucidate the mechanism of hybrid improper ferroelectric switching in this compound. By simultaneously applying an electric field and recording diffraction patterns, shifts in the intensity of superstructure peaks consistent with one of the switching mechanisms proposed by Nowadnick & Fennie [*Phys. Rev. B*, (2016), **94**, 104105] are observed. While the experiment only achieves a partial response, comparison with simulated data demonstrates a preference for a one-step switching mechanism involving an unwinding of the octahedral rotation mode in the initial stages of switching. These results represent some of the first reported experimental diffraction-based evidence for a switching mechanism in an improper ferroelectric.

## Abstract 2

This article reports the first *in situ* diffraction result collected under applied electric field on a hybrid improper ferroelectric which shows a subtle yet robust preference for a switching mechanism that proceeds via an unwinding of the octahedral rotation mode.

## 1. Introduction   

Ferroelectric materials are those which display a spontaneous electrical polarization that is switchable through the application of an external electric field. These materials are of interest because of their variety of technological applications, including sensing, electrocaloric cooling and in memory devices (Scott, 2007 ▸; Martin & Rappe, 2016 ▸). Several physical phenomena are known to drive polarization, such as second-order Jahn–Teller distortions and lone-pair ordering (Ederer & Spaldin, 2004 ▸; Von Hippel, 1950 ▸). Other distortions from a centrosymmetric structure may be achieved in the solid state through magnetic or charge ordering, or by chemical doping of the parent structure using cations of varying sizes (Rao *et al.*, 2012 ▸; Yamauchi & Barone, 2014 ▸). Regardless of the mechanism, resultant phase transitions for which the polarization is the primary order parameter (OP) generate what is known as proper ferroelectricity. For materials such as yttrium manganite, however, a structural distortion leads to corrugation of MnO_5_ layers that induces off-centre displacements of the Y^3+^ cations, resulting in a net polarization (Van Aken *et al.*, 2004 ▸). This phenomenon is known as ‘improper’ ferroelectricity, wherein the primary OP of the phase transition is not the polarization; such a mechanism was first used to explain the ferroelectricity present in gadolinium molybdate (Cross *et al.*, 1968 ▸; Levanyuk & Sannikov, 1974 ▸; Pytte, 1970 ▸).

*AB*O_3_ perovskites are a well studied class of compounds and are known to display a wide variety of physical phenomena, including giant photovoltaic effects, superconductivity, proper ferroelectricity and ferromagnetism, the latter pair of which may be coupled to form a multiferroic phase (Choi *et al.*, 2009 ▸; Bednorz & Müller, 1988 ▸; Cheong & Mostovoy, 2007 ▸; Salamon & Jaime, 2001 ▸). The related *A*_*n*+1_ *B*_*n*_O_3*n*+1_ Ruddlesden–Popper structures may be considered to consist of *n* perovskite layers separated by a rock-salt-like layer. These layered perovskites have been the subject of recent efforts to examine polarization resulting from a sub-type of the improper mechanism: hybrid improper ferroelectricity, wherein two nonpolar lattice distortions couple to a polar mode to generate an overall polar structure (Benedek *et al.*, 2012 ▸; Rondinelli & Fennie, 2012 ▸). This mechanism is of particular interest because of its potential to extend research into multiferroics by overcoming the ‘*d* ^0^ rule’, which previously limited the scope of multiferroics by disallowing magnetic *d^*n*^* cations from participating in ferro­electricity (Hill, 2000 ▸).

**Figure 1:**
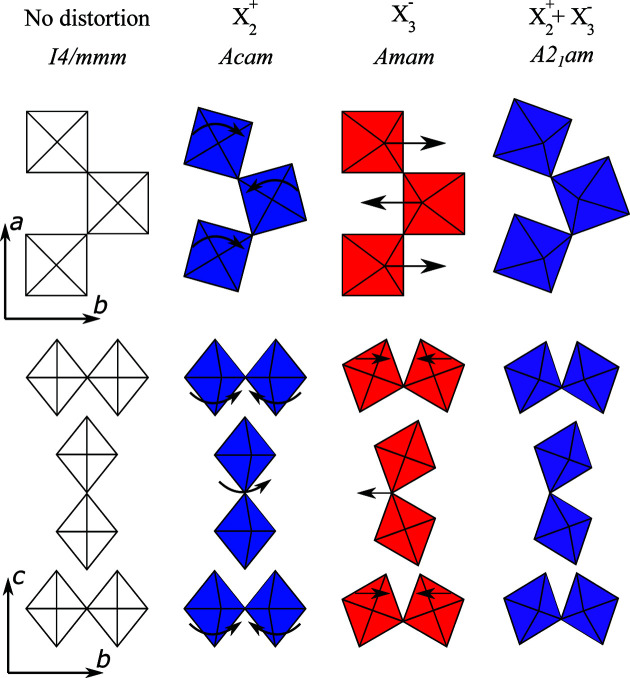
Representations of the theoretical high-symmetry *I*4/*mmm* and experimentally observed *A*2_1_ *am* structures of Ca_3_Ti_2_O_7_, with the effects of the 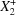 (rotation) and 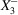 (tilt) modes highlighted.

**Figure 2:**
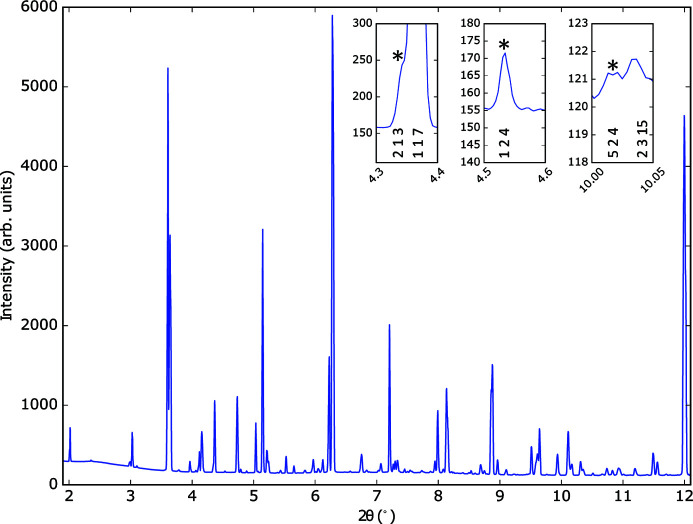
X-ray diffraction pattern of Ca_2.15_Sr_0.85_Ti_2_O_7_. Inset: selection of superstructure peaks indexed in the *A*2_1_ *am* setting (213, 124 and 524) resulting from the simultaneous condensation of 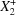 and 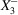 modes.

Ca_3_Mn_2_O_7_ and Ca_3−*x*_Sr_*x*_Ti_2_O_7_ (0 ≤ *x* ≤ 0.9) adopt the polar *A*2_1_ *am* space group at room temperature as a result of the hybrid improper mechanism (Benedek & Fennie, 2011 ▸). This orthorhombic structure may be considered as a distortion of the tetragonal *I*4/*mmm* aristotype by a combination of in-phase rotations and out-of-phase tilts of the TiO_6_ octahedra (Fig. 1 ▸), and corresponds to the direct sum of the symmetry spaces spanned by *Acam* and *Amam* (Senn *et al.*, 2015 ▸). The identification of this space group as opposed to the high-symmetry *I*4/*mmm* is based upon the weak superstructure peaks found at (*h* + 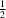, *k* + 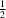, *l*). The basis of the *A*2_1_ *am* supercell with respect to the *I*4/*mmm* symmetry is (1
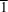
0), (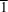 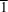
0), (00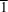), origin = (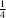, 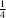, 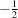) (Lobanov *et al.*, 2004 ▸). Examples of the superstructure peaks in a sample of Ca_2.15_Sr_0.85_Ti_2_O_7_ indexed in the *A*2_1_ *am* setting are shown in Fig. 2 ▸.

The rotation and tilt OPs transform as irreducible representations 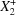 and 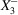, respectively, and their combination yields not only a polar ground state but also a very rich domain structure including head-to-head and tail-to-tail charged domain walls (Harris, 2011 ▸; Oh *et al.*, 2015 ▸). With increasing temperature, the phase transitions of samples of Ca_3−*x*_Sr_*x*_Ti_2_O_7_ (0 ≤ *x* ≤ 0.85) have been demonstrated to be strongly influenced by entropy: at *x* = 0, the phase exhibits a first-order transition between *A*2_1_ *am* and *Acaa*; at *x* = 0.85, a second-order phase transition between *A*2_1_ *am* and *P*4_2_/*mnm* is observed with smaller distortion magnitudes (Huang *et al.*, 2016 ▸; Kratochvilova *et al.*, 2019 ▸; Pomiro *et al.*, 2020 ▸). The continuous evolution of the OP at *x* = 0.85 is similar to that expected in a switching mechanism. The change from a first-order to a second-order phase transition occurs via a continuous decrease in the magnitude of the 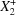 mode, followed by a rotation of the order parameter direction and a decrease in magnitude of the 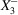 mode. For the parent *x* = 0 phase, the amplitudes of the 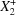 and 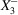 modes have been demonstrated to vary approximately linearly with respect to one another and to the polar mode between 100 and 500 K (Senn *et al.*, 2015 ▸).

In-plane piezoforce microscopy experiments performed on single-crystal samples of Ca_3−*x*_Sr_*x*_Ti_2_O_7_ have demonstrated a switchable polarization with remanent values of up to 8 µC cm^−2^, which decreased with increasing Sr substitution; similar results were found for thin-film samples (Oh *et al.*, 2015 ▸; Li *et al.*, 2017 ▸). These studies did not directly probe the octahedral distortions, instead reporting the ferroelectric behaviour of the compositions as a whole.

The evolution of the 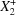 and 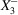 modes under the influence of an applied voltage has only been the subject of *ab initio* calculations and not experimental study. Three main pathways have been proposed as having low energy barriers: switching through a reversal in the magnitude of distortion, through an orthorhombic twin domain or through an antipolar stacking domain (Nowadnick & Fennie, 2016 ▸). To investigate the mechanism at play, we have performed synchrotron X-ray diffraction experiments on sintered pellet samples of Ca_2.15_Sr_0.85_Ti_2_O_7_, and experimental evidence that strongly favours one of the proposed theoretical mechanisms is herein presented. The experiment was performed on Sr-substituted samples as the voltage required to achieve a switch of the polarization is smaller than in the pure Ca_3_Ti_2_O_7_ phase, thus reducing the likelihood of the pellets suffering dielectric breakdown under the applied voltage.

## 2. Methods   

### 2.1. Sample preparation   

Polycrystalline samples of Ca_2.15_Sr_0.85_Ti_2_O_7_ were prepared by standard solid-state methods. CaCO_3_, SrCO_3_ and TiO_2_ were ground together in stoichiometric proportions using an agate mortar, pressed into pellets, and calcined at 1173 K for 20 h. Further heating at 1473 K for 72 h with several intermediate grinding and re-pelleting steps produced a sample of high purity. A Rietveld refinement against powder X-ray diffraction data is shown in the supplementary information (Fig. S1). The refined lattice parameters of the samples were *a* = 5.4605 (3) Å, *b* = 5.4828 (3) Å and *c* = 19.7340 (16) Å, in agreement with previously published work (Oh *et al.*, 2015 ▸). *In situ* X-ray diffraction measurements were performed at Beamline ID15A at the ESRF using a Pilatus3X CdTe 2M detector and a specialized experimental cell developed by Critus Pty Ltd (https://www.critus.com.au/) placed 1.3 m away from the detector. A photograph of the beamline arrangement and a diagram showing the sample cell are provided in Figs. S2 and S3 in the supplementary information (Vaughan *et al.*, 2020 ▸; Daniels *et al.*, 2009 ▸). A beam energy of 71.5 keV was used, equating to an X-ray wavelength of 0.17355 (25) Å (refined against a CeO_2_ standard).

A total of nine sintered pellets were cut into 2 × 1.5 × 1 mm wafers and coated on each face with conductive silver paste. Each wafer was then placed individually into the experimental cell and the upper screw electrode brought into contact with the upper surface. Samples were subjected to a voltage which followed a triangular waveform over the range 0–4 kV, cycling ten times over a period of 20, 40 or 80 s (corresponding to 0.5, 0.25 and 0.125 Hz, respectively). Complete ferroelectric switching was not achieved as sample breakdown occurred at voltages exceeding 4 kV (electric field strength 4000 V mm^−1^). We emphasize that this applied field is insufficient to achieve true switching in the sample; instead we observe the response of the individual domains to the applied field, which is indicative of the softest switching pathways.

Detector acquisition was triggered simultaneously with the initiation of the voltage such that 100 X-ray diffraction patterns were recorded, evenly spaced across the entire voltage cycle. The 80 s voltage cycles were additionally performed with a capture rate of 300 diffraction patterns over the time period, yielding a data set with a greater time resolution but reduced intensities. The wafers were moved slightly within the path of the beam between tests to allow for the potential effects of beam damage to the sample and to ensure that a wide variety of crystal grains were sampled.

### 2.2. Data processing   

The recorded diffraction patterns were radially integrated using *FIT2D* (Hammersley, 2016 ▸) and batch Pawley refinements were performed on each data set using *TOPAS Academic v6* (Coelho, 2018 ▸). The intensities of 349 individual *hkl* reflections were extracted from each refinement; by repeating this process over all XRD patterns from a single data set, the change in intensity of each reflection may be plotted with respect to time and hence with respect to the applied voltage. The results were consistent for different pellets and field parameters.

**Figure 3:**
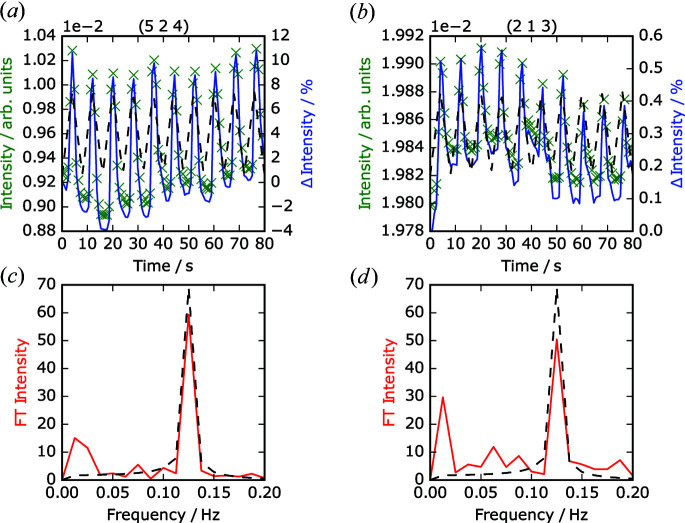
Results of time-resolved analysis for the 524 and 213 reflections. (*a*), (*b*) Plots of intensity (green crosses) and percentage change (blue solid lines); applied voltage is represented by the black dotted lines. (*c*), (*d*) Fourier transform of results in (*a*) and (*b*), showing the normalized Fourier amplitude of the reflection at 0.125 Hz (red lines) and the normalized Fourier transform of the applied field (dashed lines).

In the *A*2_1_ *am* setting, reflections with Miller indices such that *h* + *k* = odd arise from the breaking of the aristotype symmetry (*I*4/*mmm*) by the octahedral rotations and tilts. Our analysis shows that during the experiments we routinely had sensitivity to field-responsive intensity changes in a significant number of such reflections. A subset of these are listed in Table 1 ▸ (selection criteria are detailed below), with further reflections listed in the supplementary information (Table S1). Fig. 2 ▸ shows the X-ray powder diffraction pattern of Ca_2.15_Sr_0.85_Ti_2_O_7_ with some of the more clearly visible reflections highlighted. Figs. 3 ▸(*a*) and 3 ▸(*b*) show the intensity variations of the 524 and 213 reflections as a function of applied voltage.

In order to filter out reflections which showed the least response to the voltage, the data were first normalized according to equation (1): 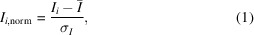
where *I*_*i*_ is the intensity associated with a reflection at a given point in time (and therefore at a given applied voltage), 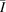 is the average intensity across the experimental run and σ_*I*_ is the standard deviation. Following this normalization, the Fourier transform of these normalized values in the time domain was calculated. The Fourier amplitude of each reflection as a function of frequency was compared, as shown in Figs. 3 ▸(*c*) and 3 ▸(*d*). A lower value of Fourier amplitude at the frequency of the applied voltage corresponds to a reflection that does not demonstrate a strong systematic response to that application of external electric field. By sorting all reflections within a data set by their Fourier amplitudes relative to the theoretical spectral weight expected for a pure single-frequency response to the applied field, the reflections which consistently demonstrate a voltage-dependent intensity shift may be identified. As an example of the result of this analytical process, we show in Figs. 3 ▸(*a*) and 3 ▸(*b*) that the intensity data for the 213 reflection displays a more noisy response to the applied voltage than that of the 524 reflection. The increased noise level results in a reduced value for the Fourier intensity at 0.125 Hz relative to the theoretical maximum and consequently a lower ranking following sorting of all reflections across all data sets. We note also that the Fourier transform of the normalized values often contains a small low-frequency value that we ascribe to extrinsic effects, probably arising from a subtle evolution of the incident beam characteristics over time due to ring current decay.

Also included in Table 1 ▸ are the relative Fourier transform amplitudes (FT amplitudes) for a selection of superstructure reflections, corresponding to the ratio of the normalized Fourier amplitude of each reflection to the theoretical maximum (*i.e.* the Fourier amplitude of the normalized applied electric field). Experimental results for the additional reflections listed in Table 1 ▸ are included in the supplementary information (Fig. S4). These reflections were selected because of their relatively large initial intensity, the wide range of 2θ values and the frequency with which they appeared with high relative FT amplitudes across multiple experimental runs. We will refer specifically to the superstructure reflections listed in Table 1 ▸ from this point forward. The consistent observation of superstructure reflection intensities varying with the applied voltage (and their high FT amplitudes according to our analysis) indicates that the experiment is sensitive to the switching process. For example, the most consistently highly ranked reflection according to our analysis is 524, fulfilling the criterion of *h* + *k* = odd.

**Figure 4:**
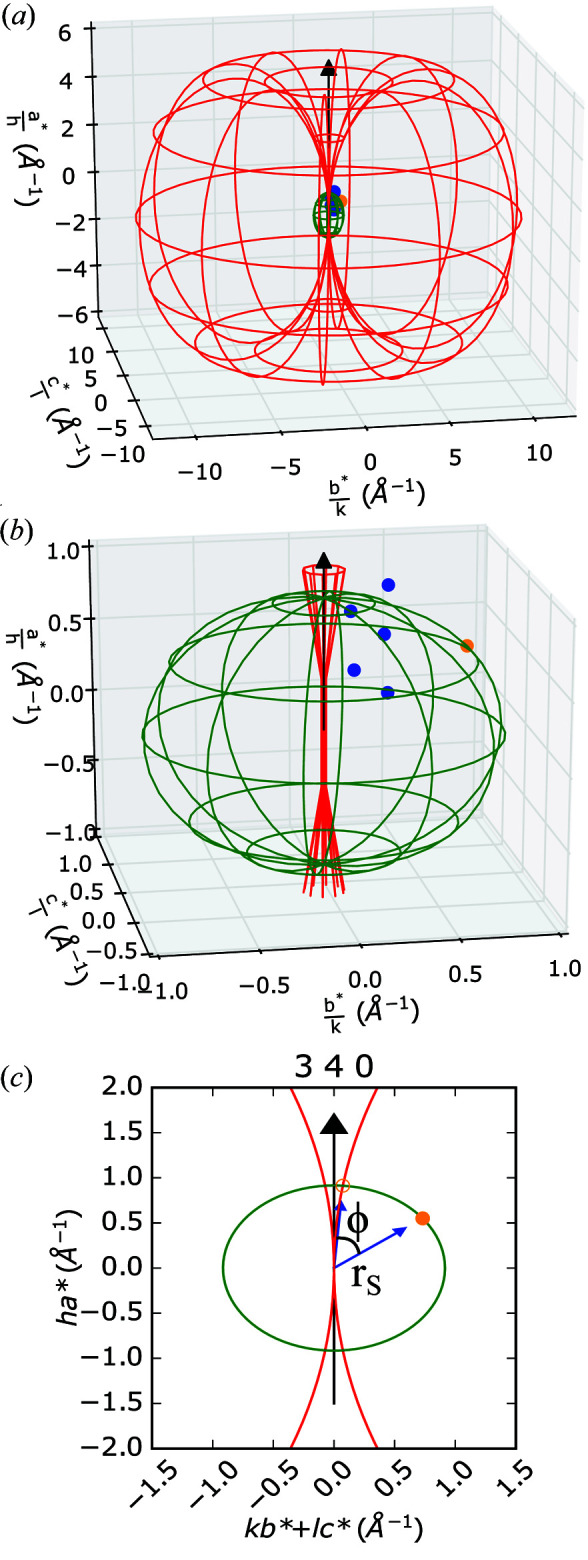
(*a*) Torus constructed using the Ewald sphere rotated about the polar a* direction with the sphere resulting from the 340 reflection; the black arrow indicates the polar direction. (*b*) Close view of the 340 sphere with the 340 reflection highlighted (orange point, other reflections from Table 1 ▸ also visible as blue points). (*c*) A simplified 2D projection of the torus, showing the angle ϕ and the radius of the reflection sphere.

Next, we consider the geometric constraints of our *in situ* switching experiment, since it is clear that not all reflections will be able to fulfil reflection conditions whilst having their polar axes optimally aligned with the external electric field. In order to understand the geometry of our experiment, we consider the symmetry constraints on the direction of polarization within the *A*2_1_ *am* space group. Each wafer sample of Ca_2.15_Sr_0.85_Ti_2_O_7_ was composed of a compressed sintered powder. Owing to the random orientation of individual crystallite grains within the wafer, the law of powder averaging results in only one-third of grains having their polar axis (the *a* axis with respect to the crystallographic setting) aligned with directions that are favourable with regard to the applied electric field. Of these, only half of the grains will be anti-aligned favourably with regard to the field and thus exhibit a switching signal in the experiment. Hence the experimental intensity change is reduced, resulting from only the one-sixth of crystallite grains that are anti-aligned with the applied field, without considering any preferred orientation present in the sample. However, the resultant component of this electric field that a grain experiences along its polar *a* axis for a given reflection condition must also be calculated by considering the Ewald sphere construction shown in Fig. 4 ▸.

Fig. 4 ▸(*a*) shows a representation of the Ewald construction for the experimental apparatus. In three dimensions, the Ewald sphere is rotated about the direction of polarization (a*) to generate a horn torus with a tube radius *r*_T_ = 1/λ, where λ is the experimental X-ray wavelength. This is the limiting Ewald torus for reflection conditions being satisfied with the constraint that the polar axis must align directly with the external field. However, since only a vanishing number of reflections will precisely fulfil this requirement, we must consider for other reflections how far the torus must be tilted relative to a* to achieve this. The cosine of this angle multiplied by the applied field then provides the resultant electric field experienced by a grain along its polar axis. It is geometrically simpler to consider a fixed torus and to tilt the reciprocal lattice about the origin. For a given reflection, the associated reciprocal lattice point carves out its own sphere with radius *r*_S_ = [(ha*)^2^ + (kb*)^2^ + (lc*)^2^]^1/2^, where *h*, *k* and *l* are Miller indices and a*, b* and c* are reciprocal lattice parameters. It is then the angle between where the torus and sphere intercept and the initial position of the reflection (prior to considering the tilting) that will provide us with the resultant electric field on the polar axis of a given grain, again by multiplying the cosine of the angle by the field. As an example, the sphere generated using the 340 reflection is shown in Fig. 4 ▸(*b*). Fig. 4 ▸(*c*) is a two-dimensional representation of this construction focusing on the reflection sphere, where a* is the vertical axis and the perspective is such that the circle projected by the reflection sphere is perpendicular to the viewer. Additional representations of this type and the complete derivation of this two-dimensional projection are shown in Figs. S5 and S6 of the supplementary information. The calculated ϕ is the angle between the vector from the reciprocal space origin to any given reflection and the vector from the origin to the intersection of the Ewald sphere with the reflection sphere; we illustrate the geometry for reflections with Miller indices greater than zero where ϕ may only take values from 0 to 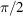. Smaller angles indicate that, for a reciprocal lattice point to be brought into the diffraction condition, only a small misalignment of the a* polar axis with the external electric field will occur. Such grains in our powder diffraction experiment will experience resultant distortions that represent the bulk of the applied electric field. For reflections with ϕ values approaching 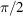, the experimental geometry effectively renders no sensitivity. It follows that our experiment is most sensitive to reflections with large *h* and small *k* and *l* values. In the subsequent analysis we hence resolve the component of the electric field onto the a* axis by 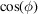. This assumption is justified since the anisotropy of the Ruddlesden–Popper structure will restrict the direction of allowed polarization. The ϕ values for selected reflections are summarized in Table 1 ▸.

## 3. Simulations   

Following the theoretical work of Nowadnick & Fennie (2016 ▸), we consider the various proposed mechanisms by which the switching may proceed. The simplest of these is the one-step switching mechanism, which corresponds to the amplitude of either of the rotation or tilt modes starting at some value *x*, passing through zero and ending at −*x* to produce the ferroelectric switching effect. However, this mechanism is predicted to have a higher activation energy to achieve complete switching than the more complex two-step mechanisms, in which the phase of the rotation and tilt modes in a single two-layer block of oxide octahedra with respect to their nearest neighbours must also be considered. Two main branches of two-step mechanisms are energetically plausible: one in which the switching proceeds *via* an antipolar space group and one in which the switching proceeds *via* an orthorhombic twinning of the octahedral blocks. As with the one-step mechanism, both of these routes may occur *via* the 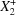 or the 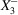 mode. To simulate the effect of rotation, tilt and polar modes upon the intensity of the reflections, distortions of the theoretical *I*4/*mmm* structure were calculated using the *ISODISTORT* program (Campbell *et al.*, 2006 ▸). By choosing different OP directions for the 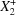 and 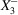 irreducible representations (‘irreps’), we generate isotropy subgroups of *I*4/*mmm* of various symmetries. For example, when the OPs are in the directions (0; *a*|0; *b*), the *I*4/*mmm* structure is transformed to *A*2_1_ *am* [basis = (00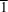), (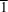 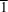
0), (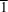
10), origin shift = (1/4, 1/4, −1/2)]. When both OPs are along general directions (*a*; *b*|*c*; *d*), the space group resulting from the distortions is *Pm* [basis = (110), (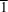
10), (001), origin shift = (0, 0, 0)]. By incorporating this lowered symmetry into a symmetry-mode model in the Rietveld refinement program *TOPAS Academic v6*, it is possible to individually vary the magnitudes and directions of the distortions and plot the effects on the intensity of each reflection. Starting with both modes fixed at values refined in a previous publication (defined as switching coordinate 0) and manipulating their amplitudes both individually and simultaneously (Pomiro *et al.*, 2020 ▸), we mimic the effect of unwinding the distortion modes (complete unwinding being switching coordinate 0.5), moving through different switching mechanisms to a complete reversal of the distortions (defined as switching coordinate 1).

## 4. Results and discussion   

**Figure 5:**
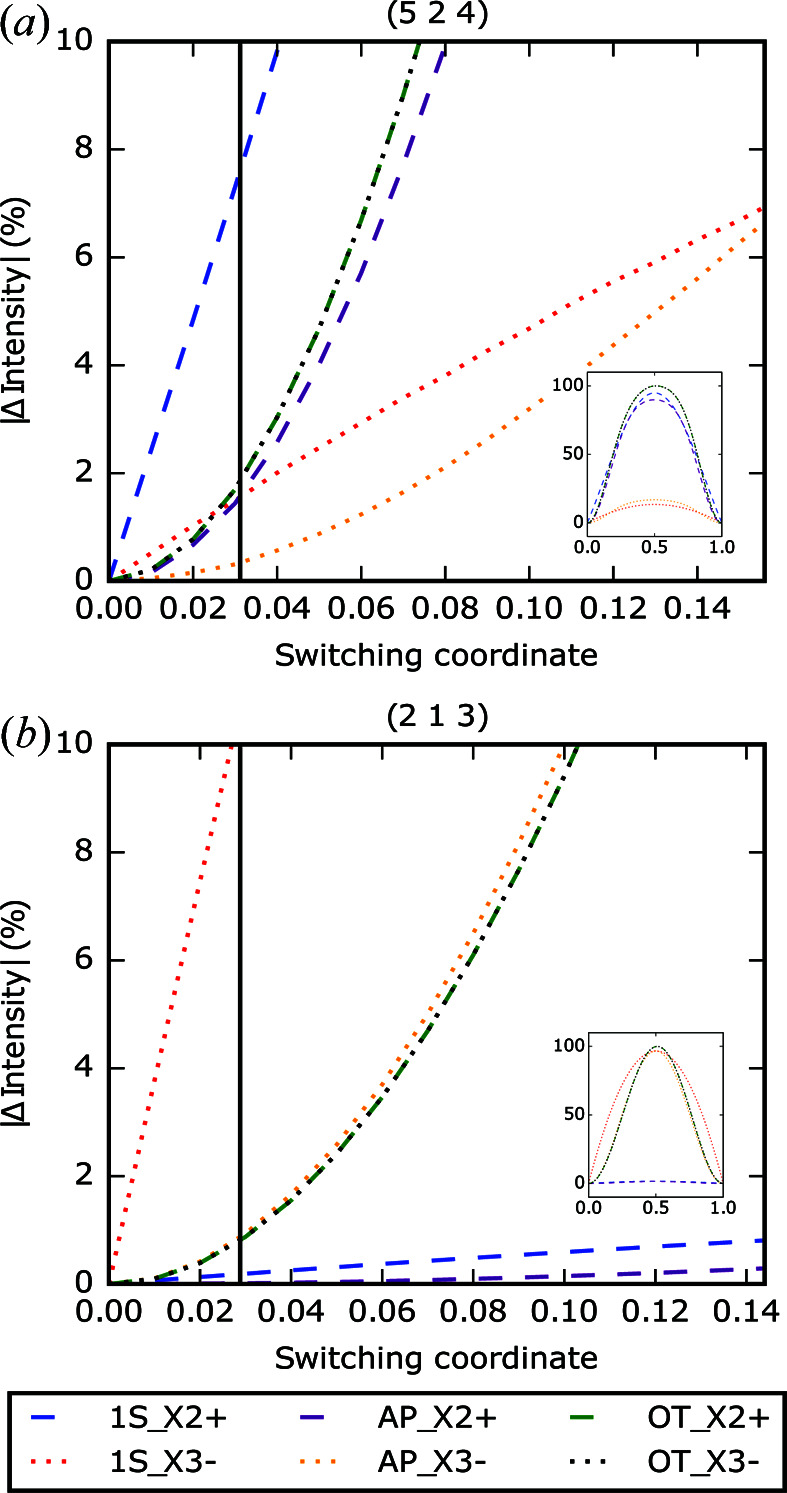
(*a*) Simulated response of the intensity of the 524 reflection under different switching mechanisms (1S: one-step mechanism; AP: two-step antipolar mechanism; OT: two-step orthorhombic twin mechanism). The upper *x* axis limit is 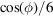 as described in the text. The black line is drawn at 20% of the upper axis limit for each reflection The inset shows the result of complete switching for all mechanisms. (*b*) Simulated response of the 213 reflection [legend and black line formatted in the same manner as (*a*)].

Fig. 5 ▸(*a*) shows the result of such a simulation for the 524 reflection. The one-step mechanism involving the 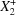 mode causes the largest change in reflection intensity over the shortest switching coordinate, changing by 10% around a switching coordinate of approximately 0.04. The next-largest-magnitude changes in intensity would be caused by either of the two-step mechanisms: switching *via* an orthorhombic twin structure and switching the 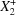 mode *via* an antipolar structure at a switching coordinate of around 0.08. Finally, the one-step and antipolar mechanisms switching the 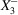 mode result in the smallest intensity change. The upper switching coordinate limit of each graph is fixed to the value of 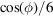 as described above, accounting for the powder averaging and misalignment of the reflection with the applied field. The two lines for the orthorhombic twin models overlap; this is a result of the initial switching processes for both mechanisms being identical. Comparing the simulated effect of the switching mechanisms with the experimental data as shown in Fig. 3 ▸(*a*), it can be seen that the observed shift in intensity of approximately 10% matches well with the one-step mechanism involving the 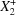 mode. While it is possible that the antipolar 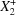 and orthorhombic twin mechanisms may also be responsible for the switching according to these data, the small progress towards achieving switching in the experiment means that the mechanism with the closest-matching behaviour over the shortest switching coordinate is the most plausible. The vertical line drawn on Fig. 5 ▸(*a*) is placed at 20% of the maximum achievable switching coordinate calculated for the 524 reflection; this indicates the approximate value of switching coordinate achieved experimentally for this reflection and shows closest agreement with the one-step 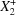 mechanism.

**Figure 6:**
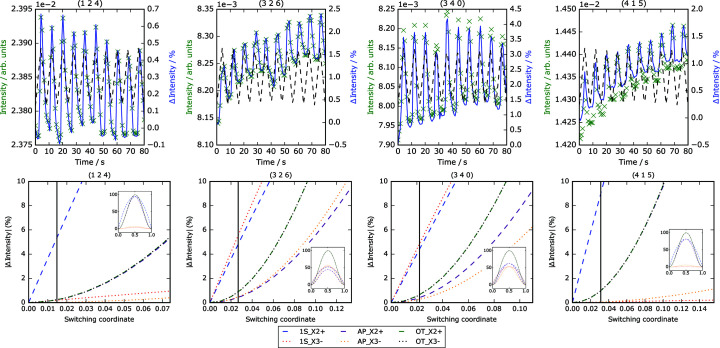
Experimental and simulated results for the 124, 326, 340 and 415 superstructure reflections (vertical lines indicate closest values of switching to fit well against experimental data). The insets show the effects of different switching mechanisms on reflection intensities at complete switching.

Fig. 5 ▸(*b*) shows the simulated effect of the different mechanisms on the 213 reflection. Comparing these simulations with the experimental data shown in Fig. 3 ▸(*b*) in the same way as for the 524 reflection, the mechanisms which most closely match the small magnitude of the observed intensity shift (around 0.5%) are the one-step and antipolar mechanisms for the 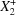 mode, with the intensity shift predicted for the one-step 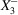 mechanism only possible at an extremely small switching coordinate. Simulations of each switching pathway on each of the remaining reflections discussed in this work are shown in Fig. 6 ▸. By applying the assumption that we have achieved around 20% of the maximum switching for each reflection shortlisted in Table 1 ▸ (a good fit for the observed intensity shifts), we qualitatively assign the most plausible switching pathway according to the analysis. Comments on these assigned mechanisms are summarized in Table 2 ▸.

We find that mechanisms which involve the 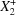 rotation mode agree more closely with the experimental data than mechanisms that involve the 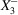 tilt mode (which are for the most part inconsistent with our observations), implying that the 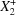 mode is the softer of the two for the present system (*x* = 0.85). However, it is difficult to draw any quantitative conclusions regarding the specific mechanism or mechanisms of action. This difficulty may be attributed to several factors, one of which is the incomplete switching achieved in our experiment. We measure here the response of the structure to the external applied field and, while this response is indicative of the softest distortion pathways in the structure, there is no evidence that even a small fraction of the aligned domains have had their direction of polarization reversed; on the contrary, the intensity of the reflections returned to their initial values when the applied field was removed. Previous computational work on this topic has found that the energy barrier to achieve a small amount of distortion from the initial *A*2_1_ *am* structure is predicted to be similar for one-step and two-step mechanisms and only becomes significantly different as the distortion progresses towards full switching (Nowadnick & Fennie, 2016 ▸). As such, if our experiment was capable of achieving higher applied fields it might be possible to observe an eventual transition to a specific mechanism at higher switching coordinate. The preferred orientation imposed by the processing of the sample into a pellet, though necessary for the experiment to proceed, also prohibits a more quantitative Rietveld-based analysis of the *in situ* data.

Another limitation of our work stems from the necessary geometric constraints of the *in situ* polycrystalline experiment. We have assumed that only 1/6 of grains experience the geometrically resolved resultant of the applied field. We also assume that the ‘switching coordinate’ function is linear in the applied field. However, grains that fulfil the diffraction condition but only experience a fraction of the resultant, such as the 124 reflection which only experiences approximately 40% of the applied 4000 V mm^−1^, may achieve much less than half the switching coordinate associated with the 524 reflection, which essentially experiences the full applied electric field. This may explain why the 124 reflection is in poorer agreement with the proposed 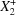 switching pathway. Also, owing to the geometric constraints of the experiment, where unswitched grains may have their polar axes either aligned or anti-aligned to the field, we are only able to compare the magnitude of any intensity changes (as opposed to resolved structural data as may be acquired from Rietveld refinement), and thus we can only obtain a qualitative estimate of the softest distortion switching pathways in the structure.

Despite these limitations, our results suggest that the initial switching pathway proceeds predominately via an unwinding of the 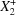 rotation mode. This is corroborated by analogy with the temperature-based phase transitions found in our previous work on this material, wherein a sequence of phase transitions from *A*2_1_ *am* to aristotype *I*4/*mmm* proceeds initially by a melting of the 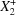 distortion (Pomiro *et al.*, 2020 ▸). We further suggest that a one-step mechanism appears to be dominant under the conditions of the experiment presented above, though the tendency towards this is subtle. It is also conceivable that several mechanisms may coexist at low switching coordinates and that a specific mechanism only dominates at higher coordinates, where the differences in energy barriers are greater.

## 5. Conclusion   

We have presented the first *in situ* X-ray diffraction experimental evidence for a particular switching pathway in Ca_2.15_Sr_0.85_Ti_2_O_7_. The experiment presented herein may be applied to further hybrid improper ferroelectric materials to advance the understanding of ferroelectric switching pathways in these systems. While it has not been considered in this work, more complex analysis may be possible by considering preferred orientation resulting from the sample morphology, or by analysing azimuthal angle dependence of the intensity changes in the 2D diffraction images.

## 6. Data availability   

Diffraction data from which the intensity modulations are extracted, and files used to generate simulations, may be accessed at https://doi.org/10.6084/m9.figshare.13607615.

## Supplementary Material

Details of superstructure reflections that showed sensitivity to the applied electric field and details of the geometric corrections applied in simulating the data. DOI: 10.1107/S1600576721001096/kc5124sup1.pdf

: https://doi.org/10.6084/m9.figshare.13607615

## Figures and Tables

**Table 1:** Shortlist of superstructure reflections used for comparisons with simulations, their 2θ values, initial intensities, maximum percentage changes in intensities, relative Fourier amplitudes and values of \cos(\phi) from Ewald sphere calculations

*hkl*	2θ (°)	*I*_0_ (10^−3^)	Δ*I* (%)	FT amplitude	cos(ϕ)
124	4.53	23.8	0.67	0.90	0.44
213	4.34	19.8	0.57	0.73	0.86
326	7.22	8.14	2.40	0.69	0.80
340	9.09	7.91	4.18	0.89	0.66
415	7.92	14.22	1.72	0.57	0.94
524	10.02	9.29	10.80	0.86	0.94

**Table 2:** Comparison of experimental data with simulated variation in intensity due to switching mechanisms and selection of most plausible mechanism according to simulations

*hkl*	Mechanism comments
124	All routes except one-step X_{2}^{+} match well, one-step X_{2}^{+} overestimates
213	One-step X_{2}^{+} and antipolar X_{2}^{+} both match well, orthorhombic twin pathway also possible
326	Both one-step mechanisms match well
340	Both one-step mechanisms match well
415	One-step X_{2}^{+} overestimates, other modes underestimate
524	One-step X_{2}^{+} matches well
