# Supplementary material for: In situ X-ray diffraction investigation of electric-field-induced switching in a hybrid improper ferroelectric
Source: J Appl Crystallogr. 2021 Mar 18;54(Pt 2):533–40. doi: 10.1107/S1600576721001096 (PMC8056766; doi:10.1107/S1600576721001096)
Supplement: Supplementary file 1 [file j-54-00533-sup1.pdf]

Support Information: In Situ X-ray Diffraction Investigation of Electric Field-Induced Switching in a Hybrid Improper Ferroelectric

Gabriel Clarke, Chris Ablitt, John Daniels, Stefano Checchia, Mark S. Senn

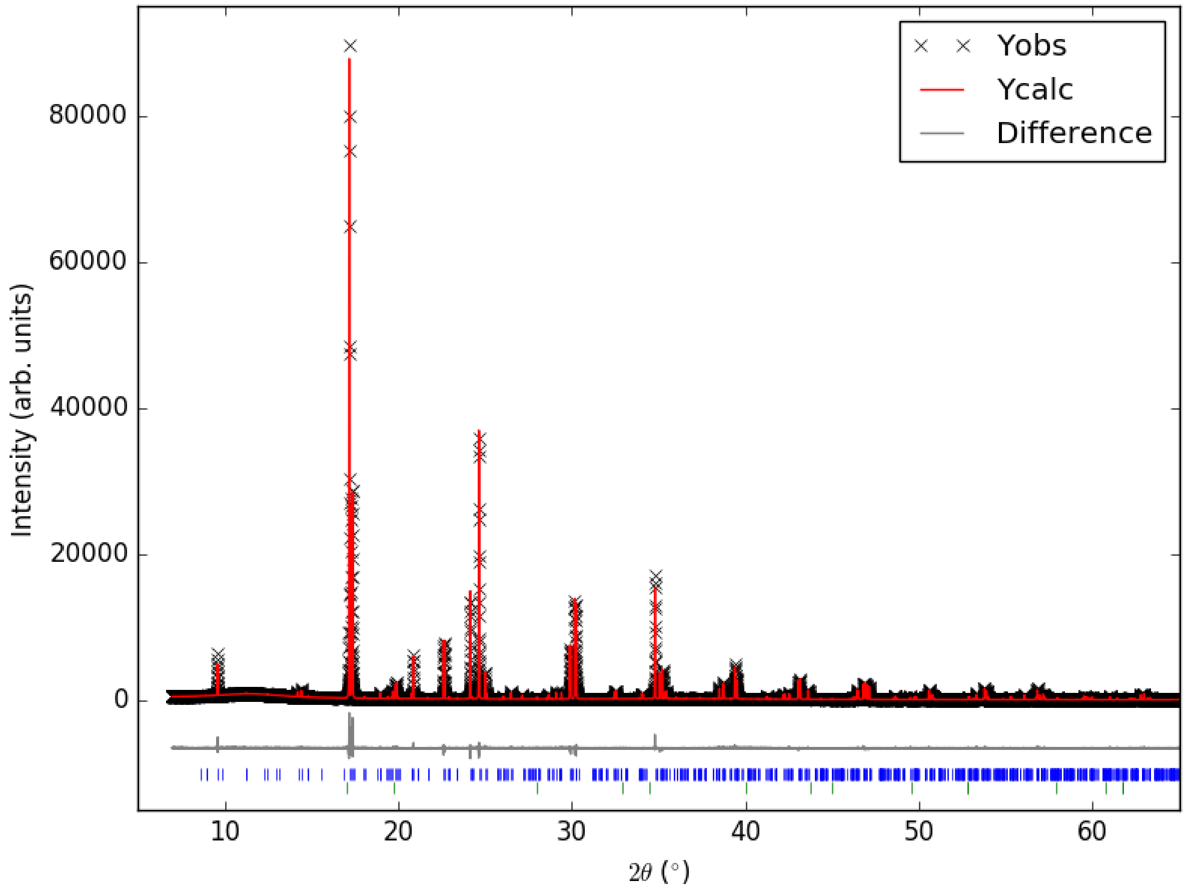

Figure S1. Rietveld refinement against synchrotron powder X-ray diffraction pattern (collected at I11, Diamond Light Source, using a wavelength of 0.82454 Å) of $Ca_{2.15}Sr_{0.85}Ti_2O_7$ sample, covering equivalent d-spacing range to Figure 2. Upper tick marks represent $Ca_{2.15}Sr_{0.85}Ti_2O_7$ reflections, lower tick marks represent reflections resulting from a small CaO impurity (approximately 0.48%)

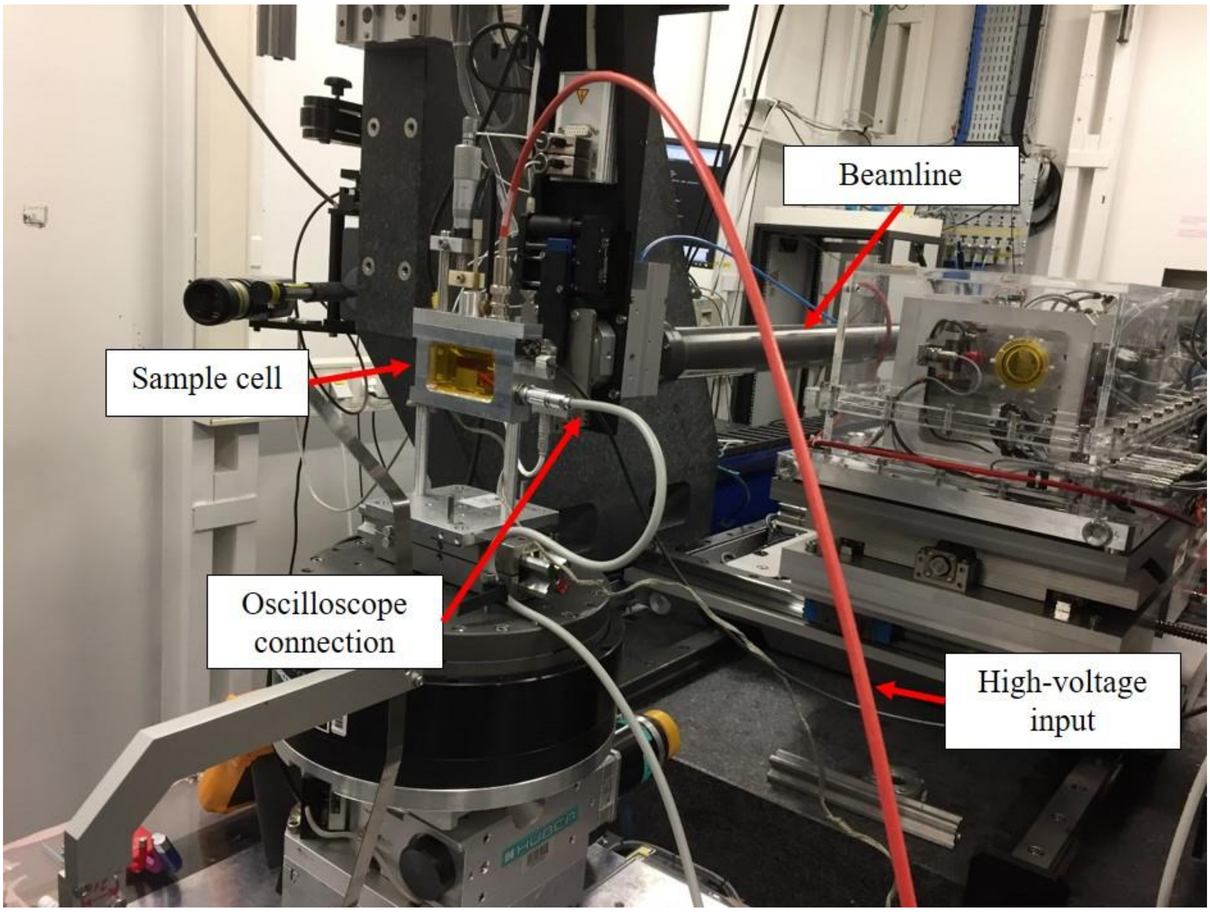

Figure S2. Photograph of sample cell on beamline

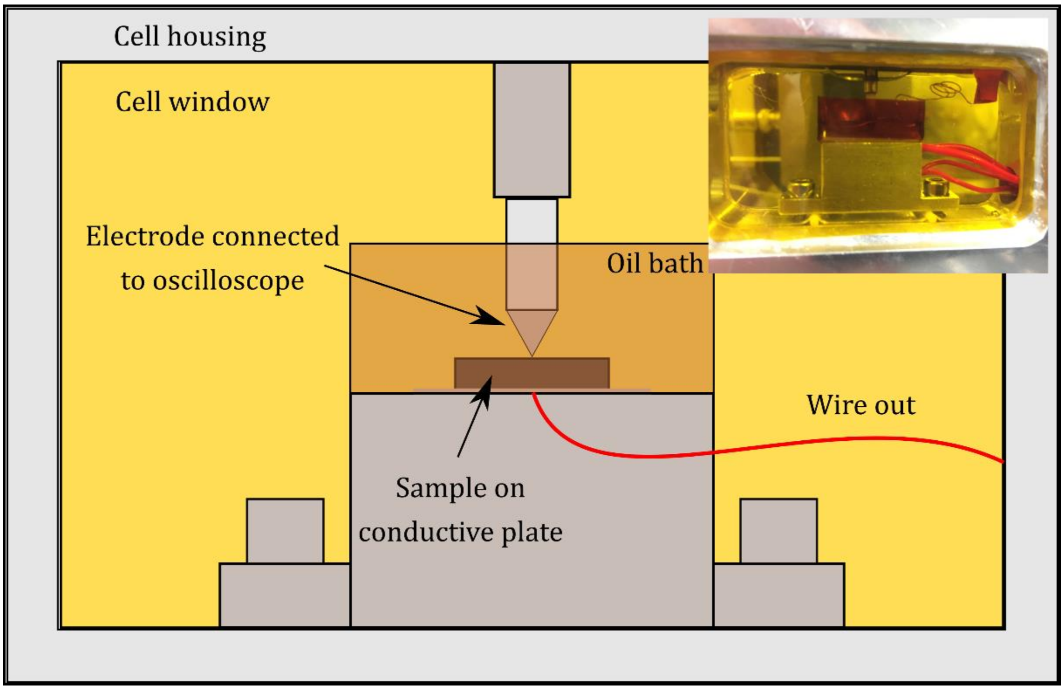

Figure S3. Diagram of sample cell

Table S1. Expanded selection of superstructure peaks consistently observed with high Fourier amplitudes at the frequency of the applied field. Overall rank, average rank and number of observations of reflection in top 50 FT rank results

Rank	Reflection	Average rank	Appearances in top 50 FT ranks across multiple runs
2	5-2-4	10	8
5	3-2-6	5.83	6
6	1-2-4	8.83	6
7	2-1-19	19.5	6
12	2-1-3	11	5
14	0-5-3	16.6	5
15	2-3-11	22.6	5
17	3-4-0	5.5	4
22	4-1-5	13	4
23	5-2-0	15.5	4
24	1-4-8	16	4
26	0-3-17	22.25	4
29	4-3-5	24.25	4
36	4-1-15	12	3
37	2-5-1	12	3
44	6-1-1	15.33	3

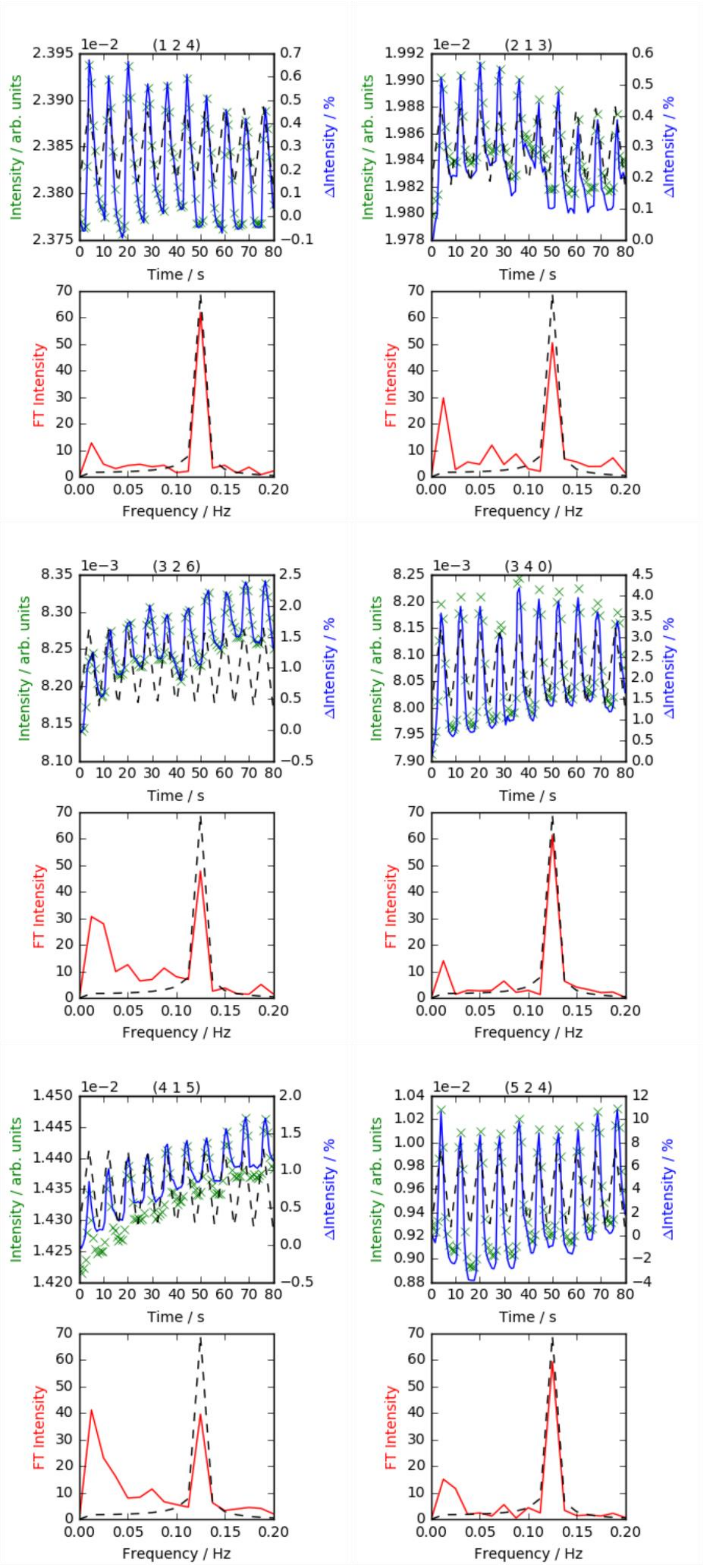

Figure S4. Experimental results for superstructure reflections (5 2 4), (3 2 6), (2 1 3), (3 4 0), (4 1 5) and (1 2 4)

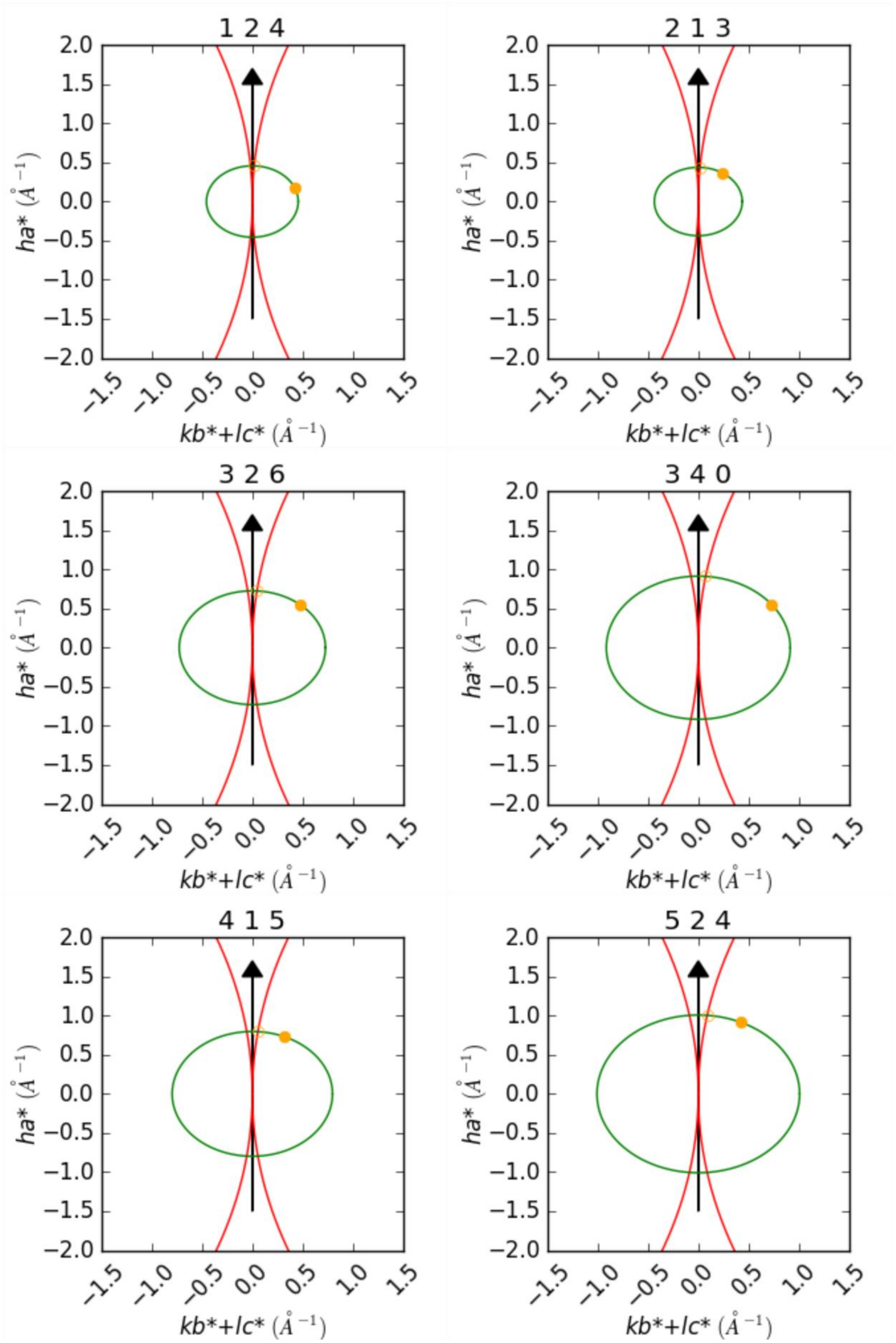

Figure S5. 2-d projections of Ewald sphere constructions for (1 2 4), (2 1 3), (3 2 6), (3 4 0), (4 1 5) and (5 2 4) reflections

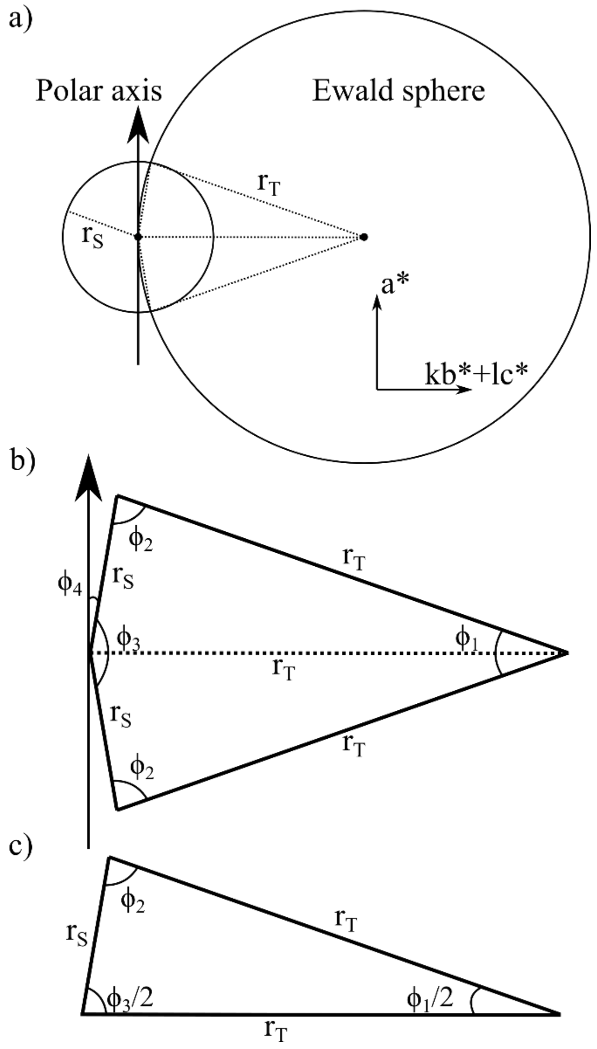

Figure S6 (a): 2-dimensional projection of Ewald sphere intersecting reflection sphere; (b): geometry of kite shape formed by radii of spheres; (c): geometry of triangle formed by bisecting kite

Geometric derivation of *hkl* intensity reduction due to Ewald sphere

In three dimensions, hkl's may be represented as points in reciprocal space with coordinates:

$$x_{hkl} = ha^*$$

$$y_{hkl} = kb^*$$

$$z_{hkl} = lc^*$$

where h, k and l denote Miller indices and a^*, b^* and c^* are reciprocal lattice parameters.

Figure S6(a) shows the two-dimensional projection with axes a^* and $kb^* + lc^*$ of the Ewald sphere and *hkl* sphere as circles of radius r_T and r_S, respectively, where:

$$r_T = \frac{1}{\lambda}$$

$$r_S = \sqrt{[(ha^*)^2 + (kb^*)^2 + (lc^*)^2]}$$

λ is the wavelength at which the experiment was performed.

The two points at which these circles intersect may be used to generate a kite of side lengths r_T and r_S containing angles ϕ_1, ϕ_2 and ϕ_3, with the angle between the polar axis and the short side ϕ_4, shown in Figure S6(b). Bisecting this kite results in the triangle shown in Figure S6(c). According to the law of cosines, the angles may be calculated according to:

$$\phi_1 = 2\cos^{-1}\left(\frac{2r_T^2 - r_S^2}{2r_T^2}\right)$$

$$\phi_2 = \cos^{-1}\left(\frac{r_S^2}{2r_S r_T}\right)$$

$$\phi_3 = 2\cos^{-1}\left(\frac{r_S^2}{2r_S r_T}\right)$$

In reciprocal space, the point at which the Ewald and *hkl* circles intersect therefore have coordinates:

$$x_{intersect} = r\cos\left(\frac{\phi_3}{2}\right)$$

$$y_{intersect} = r\sin\left(\frac{\phi_3}{2}\right)$$

The angle factor ϕ calculated in the main work is the angle between an hkl, the origin and the intersection of that hkl's sphere with the Ewald sphere.
